# Implementation of secondary fracture prevention services after hip fracture: a qualitative study using extended Normalization Process Theory

**DOI:** 10.1186/s13012-015-0243-z

**Published:** 2015-04-23

**Authors:** Sarah Drew, Andrew Judge, Carl May, Andrew Farmer, Cyrus Cooper, M Kassim Javaid, Rachael Gooberman-Hill, The REFReSH study group

**Affiliations:** Nuffield Department of Orthopaedics, Rheumatology and Musculoskeletal Sciences, Oxford NIHR Musculoskeletal Biomedical Research Unit, University of Oxford, Windmill Road, Headington, Oxford, OX3 7LD UK; MRC Lifecourse Epidemiology Unit, Southampton General Hospital, University of Southampton, Southampton, SO16 6YD UK; Faculty of Health Sciences, University of Southampton, Highfield, Southampton, SO17 1BJ UK; Nuffield Department of Primary Care Health Sciences, Radcliffe Observatory Quarter, Oxford, OX2 6GG UK; School of Clinical Sciences, University of Bristol, Learning and Research Building, Level 1, Southmead Hospital, Bristol, BS10 5NB UK

**Keywords:** Implementation, Normalization Process Theory, Qualitative research, Osteoporosis, Fragility fracture, Hip fracture

## Abstract

**Background:**

National and international guidance emphasizes the need for hospitals to have effective secondary fracture prevention services, to reduce the risk of future fractures in hip fracture patients. Variation exists in how hospitals organize these services, and there remain significant gaps in care. No research has systematically explored reasons for this to understand how to successfully implement these services. The objective of this study was to use extended Normalization Process Theory to understand how secondary fracture prevention services can be successfully implemented.

**Methods:**

Forty-three semi-structured interviews were conducted with healthcare professionals involved in delivering secondary fracture prevention within 11 hospitals that receive patients with acute hip fracture in one region in England. These included orthogeriatricians, fracture prevention nurses and service managers. Extended Normalization Process Theory was used to inform study design and analysis.

**Results:**

Extended Normalization Process Theory specifies four constructs relating to collective action in service implementation: capacity, potential, capability and contribution. The capacity of healthcare professionals to co-operate and co-ordinate their actions was achieved using dedicated fracture prevention co-ordinators to organize important processes of care. However, participants described effective communication with GPs as challenging. Individual potential and commitment to operationalize services was generally high. Shared commitments were promoted through multi-disciplinary team working, facilitated by fracture prevention co-ordinators. Healthcare professionals had capacity to deliver multiple components of services when co-ordinators ‘freed up’ time. As key agents in its intervention, fracture prevention coordinators were therefore indispensable to effective implementation.

Aside from difficulty of co-ordination with primary care, the intervention was highly workable and easily integrated into practice. Nevertheless, implementation was threatened by under-staffed and under-resourced services, lack of capacity to administer scans and poor patient access. To ensure ongoing service delivery, the contributions of healthcare professionals were shaped by planning, in multi-disciplinary team meetings, the use of clinical databases to identify patients and define the composition of clinical work and monitoring to improve clinical practice.

**Conclusions:**

Findings identify and describe elements needed to implement secondary fracture prevention services successfully. The study highlights the value of Normalization Process Theory to achieve comprehensive understanding of healthcare professionals’ experiences in enacting a complex intervention.

**Electronic supplementary material:**

The online version of this article (doi:10.1186/s13012-015-0243-z) contains supplementary material, which is available to authorized users.

## Background

Hip fractures present an important public health problem. Each year, 87,000 hip fractures occur annually in the UK [1] with a cost (including medical and social care) of around £2.3 billion a year [2]. Hip fractures usually occur when individuals with underlying osteoporosis fall [3,2]. These patients are a high risk of further fractures and premature death [4-6]. The risk of second hip fracture ranges from 2.3% to 10.6% [7,8] and mortality during the first year after fracture ranges from 8.4% to 36% [4]. Effective management of these patients can significantly reduce this risk, which is why professional bodies have produced comprehensive guidance about the management of hip fracture [1,9-12]. Fracture prevention services should have four main components: case finding those at risk of further fractures; undertaking an evidence-based osteoporosis assessment; treatment initiation in accordance with guidelines for both bone health and falls risk reduction and then strategies to monitor and improve adherence to recommended therapies [12]. Since the provision of these services is multi-disciplinary [1], guidance recommends structuring services around a dedicated co-ordinator who provides a link between all the multi-disciplinary teams involved in fracture prevention [13], an approach known as a Fracture Liaison Service [14]. However, considerable gaps in patient care following fracture still exist [15] with marked variation in how services are delivered locally [16,17], and it is unclear how best to implement these services.

The implementation of interventions is increasingly being studied using theory-led research designs [18,19]. In this study, we used extended Normalization Process Theory [20] as a theoretical framework because it characterizes and explains the *collective actions* of participants (patient, professionals and others) in the implementation of new ways of enacting and organizing health care. Such an approach is a useful counterpoint to perspectives on the transmission and stabilization of innovations through networks [21,22] and theories of individual differences in behavioural change [23,24], because it attends to the factors that promote or inhibit the kinds of co-operative work that is needed to implement service innovations in complex organizational contexts. The theory builds on previous iterations of Normalization Process Theory that have presented implementation as a social process. Extended Normalization Process Theory incorporates psychological and network perspectives into earlier iterations of Normalization Process Theory to build a more comprehensive model to explain phenomena. Previous iterations were originally used to explore professional action in the implementation of interventions in telehealth and e-health [25-27], but their application has since broadened to include a range of services across primary and secondary care [28-31]. The theories are also increasingly being applied to care settings from outside of the UK [32,33,28]. However, to our knowledge, this is the first study to use extended Normalization Process Theory.

The aim of this study was to use extended Normalization Process Theory to understand how and why secondary fracture prevention services can be successfully implemented in secondary care. This serves to inform the implementation and integration of these services into practice. In addition, as one of the first studies to use extended Normalization Process Theory, there was the potential to learn about the mechanics of its application in research.

## Methods

### Sample

A purposive sample was designed to include a range of healthcare professionals from all 11 Acute NHS Trusts in a region in England who met the criteria of working in secondary care and with experience and knowledge of secondary fracture prevention after hip fracture [34]. The sample included orthogeriatricians, fracture prevention nurses, trauma nurses, hospital practitioners in osteoporosis, surgeons and service managers.

Potential participants were identified by the clinical lead/champion in osteoporosis and an operational service manager in trauma who were both working within the region. In three waves of recruitment, the study team approached potential participants by email, followed up by reminder emails when appropriate 2 weeks later. In some cases, this was then followed up by a telephone call. We also used snowball sampling [35] such that participants recommended other healthcare professionals who they knew were also involved in fracture prevention. In total, 82 healthcare professionals were contacted to take part in the study. Of these, 43 agreed to take part. The remainder either declined or were unavailable. Rather than aiming to achieve saturation [36], the final sample size reflects our aim of conducting criterion sampling to include an appropriate range of professionals to enable us to address the research question in light of extended Normalization Process Theory and their availability [37]. This meant prioritising the recruitment of professionals most familiar with the processes of implementing fracture prevention services and ensuring participants were adequately drawn from each of the 11 hospitals.

### Ethical approval

Ethical approval was provided by the University of Oxford’s Central University Research Ethics Committee (CUREC) in 2012, reference number MSD-IDREC-C1-2012-147. Written informed consent was provided by all participants prior to interview. This confirmed that participants understood that their participation was voluntary and that they were willing to let the project researcher (SD) include anonymous quotations from them in the write up of the study. Each involved NHS Trust provided R&D approval.

### Interview procedure

A qualitative researcher (SD) undertook face-to-face interviews in 2013 with one interview conducted by telephone. Interviews were between 30 and 50 min long. A topic guide divided into two themes was used to inform interview questions (Additional file 1). Theme 1 was based on the four core elements of a fracture prevention service outlined by the International Osteoporosis Foundation (IOF) as part of the ‘Capture the Fracture’ initiative [12]. This helped the researcher to explore participants’ views on the best models of care across the four main components of a fracture prevention service and co-ordination of care. Theme 2 was structured around the four constructs of extended Normalization Process Theory to enable exploration of participants’ experiences of implementing fracture prevention services (Table 1). Participants were also encouraged to raise any issues that they thought relevant, and the interviewer used methods such as ‘probing’ to help achieve depth [38].

**Table 1:** **The four constructs of extended Normalization Process Theory**

**Construct**	**Description**
‘Capacity’	Implementing an intervention depends on participants’ capacity to co-operate and co-ordinate their actions
‘Potential’	Translating capacity into action depends on participants’ commitment to operationalize the intervention
‘Capability’	The capability of participants to enact the intervention depends on its workability and integration into everyday practice
‘Contribution’	The implementation of an intervention over time depends on participants’ contributions to enacting it by investing in meaning, commitment, effort and appraisal

### Extended Normalization Process Theory

The implementation of interventions is dependent on the ability of participants to fulfil four criteria which can be understood using four constructs [20], as outlined below:

### Data analysis

Interviews were audio-recorded, transcribed, anonymised and imported into the qualitative data analysis software NVivo. An abductive analysis [39] was conducted. This involved coding data inductively using a thematic analysis, and all data were scrutinised for relevance to implementation. To relate the data to extended Normalization Process Theory, codes were then transposed onto the four main constructs of the theory. Data was then displayed on charts using the framework approach to data organization [40]. Twenty percent of the interview transcripts were independently coded by another member of the team (RG-H), and codes compared and discussed to arrive at a single code list which was refined as the analysis progressed [41]. Descriptive accounts of the data were then generated.

## Results

### Characteristics of sample

The 43 participants comprised eight fracture prevention nurses, four orthogeriatricians, four geriatricians, two GP osteoporosis specialists, five consultant trauma orthopaedic surgeons, eight rheumatology consultants, additional nursing staff, one falls co-ordinator, one falls nurse, one bone densitometry specialist and five service managers. Between three and seven were drawn from each of the 11 hospitals. Time spent working at the hospital ranged between 1 and 27 years and years of experience since qualification in their current role ranged between 8 months to 32 years.

A summary of the codes identified and their relation to the four main constructs of extended Normalization Process Theory is outlined in Table 2.

**Table 2:** **Codes identified and their relation to the four main constructs of extended Normalization Process Theory**

**‘Capacity’**	**‘Potential’**	**‘Capability’**	**‘Contribution’**
Role of dedicated fracture prevention coordinator	High levels of support for introducing service	Fracture prevention coordinators ‘freeing up’ professionals previously engaged in care	Multi-disciplinary team meetings
Multi-disciplinary paperwork: protocols and *proforma* records	Lack of support for introducing service from some professionals	Lack of time to deliver intervention	Clinical databases
Multi-disciplinary team-work: multi-disciplinary team meetings, joint ward rounds	Relationships between different professional groups	Lack of capacity to administer DXA scans	Internal monitoring systems
Positive working relationships	Multi-disciplinary team working	Challenges faced by service users in accessing services	External monitoring systems linked to funding
Location of professionals close to the service and each other	Role of fracture prevention coordinator		
Challenge of securing co-operation and communication with GPs	Varying commitment from practitioners in primary care		
High workload in primary care impacting on time spent implementing intervention			
Written communication with GPs, especially discharge summaries and DXA reports			
Potential role of fracture prevention coordinators in primary care			

Below, we explore healthcare professionals’ experiences and views about issues that affect the implementation of services to prevent secondary fractures after hip fracture using the four constructs of extended Normalization Process Theory in more detail.

### Capacity

Implementing a fracture prevention service depends on participants’ capacity to co-operate and co-ordinate their actions.

Participants in this intervention made significant personal investments in building co-operative capacity. However, their support for the intervention was focused through the allocation of a dedicated fracture prevention co-ordinator who both organized important processes of care and supported the complex relationships between other participants. This support was manifest in two kinds of work. First, it was manifest in ‘multi-disciplinary paperwork’: the protocols and *proforma* records that gave structure to particular parts of the care process and which could be easily understood and shared between different professional groups. Second, it was present in support for multi-disciplinary teamwork that meant that discussions and decisions about policy and patients could be undertaken co-operatively and in the open.

The visibility of these two kinds of support meant that a culture of co-operation developed around the intervention. This culture depended on shared expertise, as well as belonging to a co-operative group. When professionals felt closer to the centre of the service—close to the physical location and with more involvement in implementation—then they experienced better communication and enhanced enthusiasm. A key problem for participants was therefore securing the co-operation and interest of GPs. They saw this as very variable, depending not only on the extent of the GPs experience and interest in fracture prevention but also on structural factors to do with primary care workload and reimbursement. Responding to these perceived difficulties involved attempting to improve written communications—especially discharge summaries and dual energy x-ray absorptiometry (DXA) reports measuring bone density. Participants invested a good deal of effort in these activities but recognized that they were not necessarily the most effective mechanism for informing GPs. They saw a solution in extending the fracture prevention co-ordinator role into the community through a cadre of specialist nurses that could work with GPs to improve the quality of clinical care (Additional file 2).

### Potential

Translating capacity into action depends on participants’ commitment to operationalize the fracture prevention service.

Participants in this study were mainly enthusiastic and committed to enacting the intervention. They agreed that the fracture prevention service was necessary, and they were highly supportive of the service in action. Those that were not tended to be characterized by their peers as negative or unsupportive personalities, but it was clear that this lack of support—sometimes manifest in open obstruction from a small number of clinicians—was a problem that reflected other longstanding complexities in relations between different specialists and within hospital departments. These individual differences are common problems but did not present insuperable difficulties and were overcome because of the shared commitments that developed through co-operative and multi-disciplinary working that the service seemed to inspire. However, that multi-disciplinary culture of co-operation needed to be built and maintained. Some participants were clear that responsibility for sustaining shared commitments was owned by the fracture prevention co-ordinator and that a central part of the co-ordinator’s role was to glue together different professional cultures and ensure that there was constant agreement between them about the goals of the service. Once again, this meant that there were complexities at the margins and that the perceived divide between the hospital service and primary care was a very visible one, and GPs were seen as disengaged from the work of fracture prevention (Additional file 3).

### Capability

The capability of users to enact the components of a fracture prevention service depends on their potential for workability and integration in their everyday practice.

We have already emphasised that participants were enthusiastic about introducing the fracture prevention service because it introduced dedicated specialist nurses who delivered a new layer of service provision that centred on co-ordination and support, but at the same time, freed up the capacity of others who had previously been engaged in this work. In this sense, the service intervention was apparently highly workable and easily integrated into practice. But there were also important problems. These focused on three kinds of inequality. First, there were inequalities of attention. Participants characterized the settings in which they worked as under-staffed and under-resourced, and this reduced the time that could be committed to patients but also, and just as important, to do the administrative and communications work that is necessary to ensure the effective operation of a clinical service. Second, there were inequalities of equipment. Participants pointed to the lack of capacity to administer DXA scans that arose from a small number of scanners operated by equally small numbers of staff. Finally, there were inequalities of patient access, participants pointed to the difficulties that some service users faced, because of poor coverage of public transport that some socio-economically disadvantaged users needed to get to the service in the first place (Additional file 4).

### Contribution

Participants’ contributions to enacting a fracture prevention service depend on them investing in meaning, commitment, effort and appraisal.

It is important to note that the introduction of a fracture prevention co-ordinator into these clinical services did not change the content of clinical work, but rather aimed to change its organizational structure and service delivery. We have discussed above how this seemed to involve important cultural changes in relations between different professions and specialisms, but it also involved some important changes in the ways that these groups made sense of fractures as an operational problem.

The introduction of a co-ordinator effected the explicit differentiation not only of the task of organization but also of the new ways to respond to it. Planning—through multi-disciplinary team meetings—seems to have become an important mechanism by which health professionals turned their potential and capacity to deliver the new service configuration into a coherent process and enrolled others into it. Clinical databases of different kinds were an important tool through which this was accomplished, since they specified individual beneficiaries of the service but also defined the level and composition of the whole clinical workload. Poor quality data was an obstacle to this, not simply because it interfered with identifying candidate patients and delivering clinical practice, but because it interfered with monitoring the outcomes of the work, both for individual patients and for healthcare providers. Monitoring was central to the enterprise and took two forms. First, it involved understanding clinical activity as a dynamic process within the hospital, attempting to understand the degree of continued bone protection therapy after patient discharge and attempting to appraise patient compliance with those therapies. Second, it involved linking clinical activity to funding mechanisms—whether this was the Best Practice Tariff in hospitals (where it seemed to deliver significant financial benefits) or the Quality Outcomes Framework in primary care (where it did not). Here, monitoring was seen as an important mechanism to bring about change, because it focused on the dynamic performance of service providers and the degree to which they could operationalize their shared goals (Additional file 5).

## Discussion

### Principal findings

This study has identified and explored healthcare professionals’ experiences and views about issues that affect the implementation of secondary fracture prevention services using extended Normalization Process Theory [20]. Results showed that health professionals’ capacity to co-operate and co-ordinate their actions were achieved by using dedicated fracture prevention co-ordinators who organized important processes of care. Effective communication with GPs was seen as challenging and participants advocated improvements in written communication whilst recognising that this had its limitations. The potential and commitment of health professionals to operationalize secondary fracture prevention services was generally high. Shared commitment to operationalisation was promoted through multi-disciplinary team working, facilitated by fracture prevention co-ordinators. However, healthcare professionals in secondary care saw the commitments of GPs as more variable. Health professionals’ capability to enact the components of fracture prevention services was enhanced by the presence of fracture prevention co-ordinators who ‘freed up’ other healthcare professionals. As key agents in its intervention, fracture prevention coordinators were therefore indispensable to effective implementation. Aside from the challenges of cooperation and communication with primary care, fracture prevention services were seen as highly workable and easily integrated into working practice. Nevertheless, inequalities in attention to patients, equipment and patient access posed serious threats to successful implementation. To deliver the service over time, health professionals’ contributions were shaped by effective planning in multi-disciplinary team meetings, the use of clinical databases to accomplish patient care and monitoring to improve clinical practice.

### Relationship to current literature

This study contributes to the existing body of work to identify the best models of care for the prevention of secondary fractures after hip fracture by exploring how these services are best implemented in practice. Previous research has highlighted the challenge of coordinating the multi-disciplinary teams involved in fracture prevention, and there is an international consensus about the need for co-ordinator-based models of care [12,13,42-44]. To promote the integration of primary and secondary care, the British Orthopaedic Association recommends ensuring that GPs receive comprehensive discharge summaries and recommendations alongside introduction of fracture prevention champions into primary care [1]. These strategies were also advocated by participants in the study described here. The potential value of introducing clinical databases and collecting and utilising audit data and cost-saving incentives has also been highlighted [44]. This study provides evidence of how these recommendations may be implemented in working practice within the NHS. In addition, the study identifies and describes the importance of potential and commitment of healthcare professionals to operationalize fracture prevention services. It also highlights the value of fracture prevention co-ordinators, not just in coordinating and organizing care but also in sustaining the shared commitments of the multi-disciplinary team.

Findings from this study can be understood within the wider context of the NHS. A lack of capacity for healthcare professionals to co-operate and co-ordinate their actions, especially across primary and secondary care, is apparent in a number of other conditions [45-47] and remains a persistent problem for policy makers [48]. In addition, there are claims that inequalities in attention to patients, equipment and patient access exist across the NHS [49]. Recent efficiency savings may present a further challenge to this [50].

### Strengths and weaknesses of the study

The use of qualitative methods enabled us to undertake a detailed exploration of the opinions and experiences of professionals. The sample size of 43 participants was relatively small given the variety of professions included in the study, but we are confident that the data generated was sufficient to provide appropriate and detailed analysis of the experience and views of professionals working in secondary care settings. We do not distinguish between the views of different groups of healthcare professionals working within the field of fracture prevention as we found that there was little difference in their perspectives. Within the interviews, participants were asked to describe previous experiences of service implementation, and such recall may be influenced by memory and subsequent experience. Participants were also asked to define reasons for delivery of services, and this means that they would refer to their views about the practice of others. This type of data does not incorporate the perspectives of those other health professionals. This particularly applies to views about primary care, and participants were reflecting on their experiences of interaction with primary care. Also, the study only took place in one region, and although it included all NHS Trusts in that region, further work may be needed to explore fracture prevention services in other areas. We invited all health professionals (*n* = 82) involved in service delivery to take part in the interviews. Of these, 43 took part. A potential limitation of the study is that those who declined participation were less amenable or involved in intervention delivery, but the study nonetheless garnered much information about positive and negative aspects of implementation from those professionals who took part.

The theory was found to be valuable in helping us to explore the research topic because the theory was able to account for all of the experiences and challenges encountered by healthcare professionals in implementing the intervention. A challenge in the application of extended Normalization Process Theory was the overlapping nature of the constructs, meaning that data could be coded into more than one construct. A decision was therefore made to code data into more than one construct where relevant. In addition, we sometimes found it hard to be certain that we were categorising data into the ‘correct’ construct. Both of these issues are consistent with the existing literature [28]. To mitigate this, the study researchers collaborated closely with each other throughout the process to make decisions about how to code the data, to arrive at an agreed code list and application of the list. There was also the potential for tension between undertaking an abductive approach [39] whilst ensuring the data was not ‘forced’ into pre-defined constructs. Coding the data inductively using a thematic analysis before transposing it onto the constructs of extended Normalization Process Theory helped to address this since we first inductively coded and scrutinised all data for issues relating to implementation before applying extended Normalization Process Theory.

### Further research

As the study is focused solely on the perspectives of professionals working in secondary care, further work could explore experiences of engagement with fracture prevention services and service provision in primary care. This would offer a comprehensive, ‘system-wide’ perspective which would over arch the division between primary and secondary care. Further research could also explore the experiences of hip fracture patients and their significant others of accessing these services to add a ‘patient centred’ context to the implementation of these services. In addition, whilst the study focused on fracture prevention rather than falls prevention services, we acknowledge that these are interrelated [1] and further studies could employ implementation science to explore these in complement to this study.

## Conclusions

The study has successfully used extended Normalization Process Theory [20] to analyse the experiences of healthcare professionals when they work to enact a complex intervention. Identifying issues that impact on the implementation of facture prevention services after hip fracture provides information to healthcare professionals and service managers on how best to implement services for patients in the future. In addition, by using the most recent iteration of Normalization Process Theory [20], this study builds the evidence base that makes use of implementation science.

## Additional files

Additional file 1:
**Interview topic guide: themes and subthemes explored in the interviews.**
Additional file 2:
**Capacity.**
Additional file 3:
**Potential.**
Additional file 4:
**Capability.**
Additional file 5:
**Contribution.**
