# Supplementary material for: Implementation of secondary fracture prevention services after hip fracture: a qualitative study using extended Normalization Process Theory
Source: Implement Sci. 2015 Apr 23;10:57. doi: 10.1186/s13012-015-0243-z (PMC4470053; doi:10.1186/s13012-015-0243-z)
Supplement: Additional file 1: — Interview topic guide: themes and subthemes explored in the interviews. [file 13012_2015_243_MOESM1_ESM.pdf]

Additional file 1: Interview topic guide: themes and subthemes explored in the interviews

Theme 1

Current service provision – things that work well/ not so well/ improvements/ best way of doing this

- Case finding or identifying those at risk
- Assessing patients 'at risk'
- Treating patients
- Monitoring patients

Co-ordination of care - things that work well/ not so well/ improvements/ best way of doing this

- Within hospital services
- With primary care services

Theme 2

Change in services - Most significant change in how services to prevent secondary fractures after hip fracture were delivered whilst at the hospital

- How easy/ difficult process was

Capability

- Change in work done
- Access to resources
    - Finances, staffing, technology

Capacity

- How well everyone communicated with each other during the process
- How well everyone worked together to introduce the service

Potential

- How useful YOU thought the service was that was being introduced/ how useful you thought other colleagues felt about the service was that was being introduced

Contribution

- Collection of outcomes data
