# Supplementary material for: Implementation of secondary fracture prevention services after hip fracture: a qualitative study using extended Normalization Process Theory
Source: Implement Sci. 2015 Apr 23;10:57. doi: 10.1186/s13012-015-0243-z (PMC4470053; doi:10.1186/s13012-015-0243-z)
Supplement: Additional file 2: — Capacity. [file 13012_2015_243_MOESM2_ESM.pdf]

Additional file 2: Capacity

[As the fracture prevention co-ordinator] I'm the key link in it all to be honest; it's very much me who kind of sits in the middle really. I will link out to anybody any service that the patient needs. [Participant ID: 002]

[The fracture prevention team] have a separate Monday morning meeting as well to discuss every patient individually. So I think because of that there is a lot of communication both verbally and written as well, so I don't think we have any issues there at all. [Participant ID: 003 C]

The meetings that we attend [are useful as] you kind of gain a mutual professional respect. [Participant ID: 004]

There's not been a huge amount of engagement with primary care… they seem to be very – two separate camps: there's what actually happens in trauma and then there's what happens in primary care, and the communication is difficult [Participant ID: 009]

GPs get probably 400 or 500 letters a day, do they read everything? Hopefully they do. [Participant ID: 035]

GPs are fantastic, but how can they be experts and know everything… And that's why I think we have a duty to them and to our patients to inform appropriately. [Participant ID: 042]
