# Supplementary material for: Implementation of secondary fracture prevention services after hip fracture: a qualitative study using extended Normalization Process Theory
Source: Implement Sci. 2015 Apr 23;10:57. doi: 10.1186/s13012-015-0243-z (PMC4470053; doi:10.1186/s13012-015-0243-z)
Supplement: Additional file 3: — Potential. [file 13012_2015_243_MOESM3_ESM.pdf]

Additional file 3: Potential

I mean we were so desperate for it that we bent over backwards to make it work. I mean it was never a problem in the sense that if it's a service you've been asking for a very long time, if someone finally provides it you're going to make sure it works and give that individual as much support as they need to come into the system. [Participant ID: 001]

So there's some people who historically are the ones that are resistant to change are just dissatisfied, disgruntled, have lost enthusiasm, just mistrust, have become jaded with the system… Sometimes it can be very obstructive [Participant ID: 006]

I think those particular individuals probably looked down on geriatricians and so they didn't like it that a geriatrician was coming and saying… this is when you operate and they didn't like being told what to do [Participant ID: 005]

It's just about encouraging people to know that they all have a role, we all have a responsibility to deliver the quality care and all of us are important in making that so. You know each of you can't do it without the other and it's actually about ownership and responsibility [Participant ID: 026]

It needs a strong lead, it needs a me or equivalent of me really… In terms of the Orthopaedic Team, you know you're working with Orthopaedic Nurses so it's a different culture set… So you need to sort of pull in the ethos [Participant ID: 026]

I think the amount of interest from General Practice is variable and I think the variability to be honest on the whole is average if you're lucky. And I think there isn't an engagement I don't feel, in the majority of Primary Care, and ownership of secondary prevention fracture. [Participant ID: 027]
