# Supplementary material for: Implementation of secondary fracture prevention services after hip fracture: a qualitative study using extended Normalization Process Theory
Source: Implement Sci. 2015 Apr 23;10:57. doi: 10.1186/s13012-015-0243-z (PMC4470053; doi:10.1186/s13012-015-0243-z)
Supplement: Additional file 4: — Capability. [file 13012_2015_243_MOESM4_ESM.pdf]

Additional file 4: Capability

[The introduction of the service was not that difficult because] the model is that [the fracture prevention co-ordinators] just come in and do everything. So we're not asking other people to do much else for hip fracture…. We're not even asking the trauma team to identify the hip fractures because the fracture liaison service is basically doing everything. [Participant ID: 007]

I think there are obviously different demands on people's time which sometimes means care is delayed [Participant ID: 018]

There is one Orthogeriatrician who does all that work but of course she can't be here 24 hours a day, seven days a week so there have been some patients who will occasionally come through and slip through the net. [Participant ID: 001]

We do at least 3,500 scans a year with one scanner and two people… We have to meet the Government targets, the six week diagnostic targets and we are just about making those targets with difficulty. [Participant ID: 025]

Our biggest barrier is obviously the fact that we have to drag our patients from [another town] down to [the city]… So I guess it's a demographics and the location in relation of distance travelled, that means sometimes people are reluctant [to attend]. [Participant ID: 004]
