# Supplementary material for: Implementation of secondary fracture prevention services after hip fracture: a qualitative study using extended Normalization Process Theory
Source: Implement Sci. 2015 Apr 23;10:57. doi: 10.1186/s13012-015-0243-z (PMC4470053; doi:10.1186/s13012-015-0243-z)
Supplement: Additional file 5: — Contribution. [file 13012_2015_243_MOESM5_ESM.pdf]

Additional file 5: Contribution

I can modify [the guidelines for assessment] how I want to. You know because it doesn't work perfectly at the beginning and things change and you know the hospital and the Government introduce new requirements so you have to modify your form [Participant ID: 017]

It depends which doctors have been on the night before as to how much has been put onto [the computer system]… some of it turns out to be rubbish [Participant ID: 010]

[Auditing] it would be helpful because if you know what your compliance rate is you know whether or not you're having an impact. So if you've got a service whereby you're suggesting that they have a treatment and then it turns out that no one actually takes these, well then why are you wasting your resources on trying to you know. [Participant ID: 024]

We have a score card so we can get all the compartments of the Best Practice Tariff… so we keep a track of what things are, which things are going well, which things we are not performing very well and obviously look for the reasons for any shortcomings [Participant ID: 012].
